# Malignancy Rate of Bethesda Class III Thyroid Nodules Based on the Presence of Chronic Lymphocytic Thyroiditis in Surgical Patients

**DOI:** 10.3389/fendo.2021.745395

**Published:** 2021-09-30

**Authors:** Yoon Young Cho, Yun Jae Chung, Hee Sung Kim

**Affiliations:** ^1^ Division of Endocrinology and Metabolism, Department of Medicine, Soonchunhyang University Bucheon Hospital, College of Medicine, Soonchunhyang University, Bucheon, South Korea; ^2^ Division of Endocrinology and Metabolism, Department of Internal Medicine, College of Medicine, Chung-Ang University, Seoul, South Korea; ^3^ Department of Pathology, College of Medicine, Chung-Ang University, Seoul, South Korea

**Keywords:** atypia of undetermined significance, fine-needle aspiration, chronic lymphocytic thyroiditis, Hashimoto’s thyroiditis, thyroid malignancy

## Abstract

**Background:**

Hashimoto’s thyroiditis (HT), also known as chronic lymphocytic thyroiditis (CLT), may interfere with the accurate cytological diagnosis of thyroid nodules. Recently, HT has been considered a premalignant condition for thyroid cancer development. The diagnosis of atypia of undetermined significance/follicular lesions of undetermined significance (AUS/FLUS) thyroid nodules is challenging and evidence for the malignancy risk of AUS/FLUS thyroid nodules coexisting with CLT is scarce. Therefore, we assessed the malignancy risk of AUS/FLUS thyroid nodules according to the presence of background CLT.

**Methods:**

This study included 357 surgically resected thyroid nodules with AUS/FLUS cytology. Cases with concomitant malignant nodules were excluded. CLT was defined based on the pathologic report after thyroid surgery.

**Results:**

Among 357 tumors, 130 tumors (36%) were confirmed to have coexisting CLT, and 170 tumors (48%) were determined to be malignant after thyroidectomy. Malignancy rates were similar in both groups (48% in each) regardless of background CLT (62/130 with CLT *vs.* 108/227 without CLT). In the group with CLT, thyroiditis was more frequent in the final pathology (12% with CLT *vs.* 1% without CLT, P = 0.003). In multivariate analysis, positive *BRAF* ^V600E^ mutation, highly suspicious sonographic features (K-TIRADS 5), and smaller thyroid nodules were significant factors for thyroid malignancies.

**Conclusion:**

The malignancy rate of thyroid nodules with AUS/FLUS cytology was comparable irrespective of the presence of underlying CLT.

## Introduction

Thyroid nodules are the most prevalent endocrine disease and are detected in approximately 50% of the adult population when using high-resolution ultrasonography (US) (1, 2). US is the initial diagnostic approach of choice to characterize thyroid nodules. US-guided fine-needle aspiration (FNA) cytology (FNAC) categorizes most thyroid nodules as benign (Bethesda class II, 60–70% of cases) or malignant (Bethesda class VI, approximately 5% of cases) (3, 4). However, approximately 20–30% of cases fall into indeterminate (Bethesda classes III, IV, and V) categories based on FNAC (3, 4). To avoid unnecessary surgery for thyroid nodules with indeterminate cytology, clinical and sonographic features and investigations such as repeated FNA or molecular testing are used to estimate the malignancy risk (4). Unlike the implied malignancy risk (5–15%) of Bethesda class III thyroid nodules in the general population, the malignancy risk increases up to 50–60% in surgically resected populations (5–7). Bethesda class III is a heterogeneous category that contains follicular cells exhibiting either architectural abnormalities or nuclear atypia (3). Thus, this category is challenging in terms of both diagnosis and management.

Hashimoto’s thyroiditis (HT), also known as chronic lymphocytic thyroiditis (CLT), interfere with the accurate cytological diagnosis of thyroid nodules (8), although CLT is diagnosed based on the pathologic findings in principle. CLT is characterized with enlarged nuclei as well as lymphocytic and plasma cell infiltration, and lesions of CLT vary in intensity from one part of the gland to another, which may complicate the preoperative diagnosis of thyroid nodules (3, 9). In a recent study by Mulder et al. (10), a lower incidence of malignancy in AUS/FLUS thyroid nodules coexisting with CLT compared with AUS/FLUS nodules without CLT was reported. However, patients with HT have a 2-fold higher risk of developing papillary thyroid carcinoma (PTC) than patients with thyroid nodules without HT (11). In particular, in studies conducted in Asia (odds ratio (OR) 2.79, 95% confidence interval, CI 2.15–3.61), a higher risk of developing PTC in HT populations was observed compared with HT populations from Europe and the USA (OR 1.56 and 1.92, respectively) (11).

Evidence for the malignancy risk of AUS/FLUS thyroid nodules coexisting with CLT is scarce, and the risk might be geographically different based on the nutritive condition of iodine. Therefore, the malignancy risk of AUS/FLUS thyroid nodules based on the presence of CLT was assessed in the present study using surgically resected specimens from Korean subjects residing in iodine-sufficient areas.

## Materials and Methods

### Subjects

**Figure 1:**
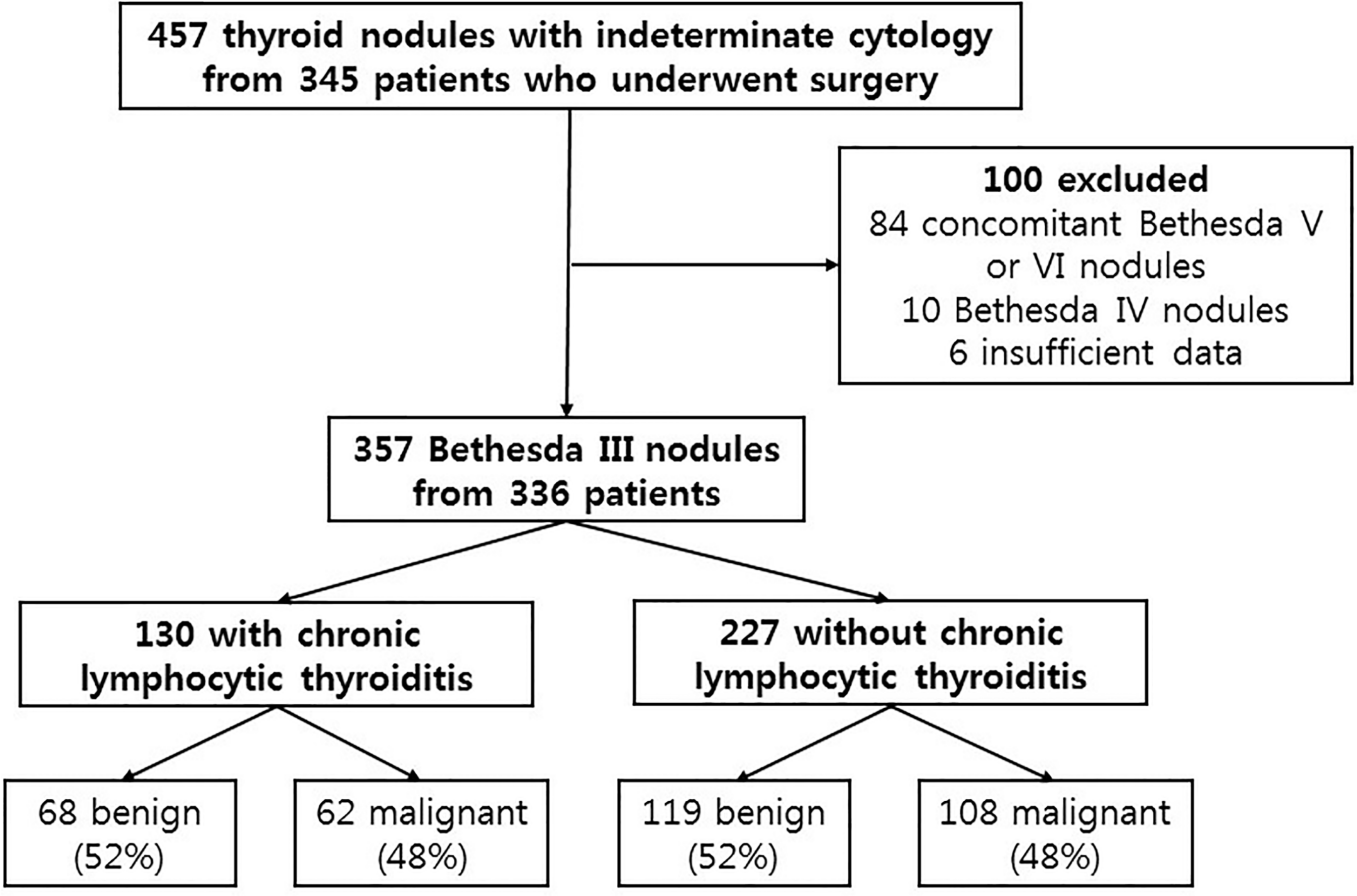
Schematic presentation of the study design.

The present study included 357 thyroid nodules with AUS/FLUS cytology from 336 patients who underwent thyroid surgery; the first FNAC was conducted between February 2013 and August 2020 at Chung-Ang University (**Figure 1**). Initially, a total of 457 indeterminate thyroid nodules from 345 patients were reviewed for this study. However, 84 cases with concomitant nodules with Bethesda class V or VI were excluded because approximately 30% of PTCs have multifocal tumors (4), which leads to overestimation of the malignancy rate of AUS/FLUS cytology. In addition, 10 cases with Bethesda class IV and 6 cases lacking data were excluded.

Medical records were retrospectively reviewed for patient age, sex, BMI, sonographic findings for thyroid nodules, cytological and pathologic results, extent of thyroid surgery, thyroid function, thyroid autoantibody level at the time of the initial FNAC, and medication for thyroid diseases. The Institutional Review Board of Chung-Ang University Hospital approved the study protocol. Patient approval and informed consent for the retrospective review of US images and medical records were waived.

### Sonographic Evaluation

Thyroid nodules were categorized according to the Korean Thyroid Imaging Reporting and Data System (K-TIRADS) to assess the malignancy risk stratification of thyroid nodules proposed by The Korean Society of Thyroid Radiology (12). K-TIRADS is a pattern-based qualitative system defining four categories (benign, low suspicion, intermediate suspicion, and high suspicion) with different risks of malignancy and has shown excellent diagnostic performance in the examination of thyroid nodules (13, 14).

### Cytology and Pathology Review

Routine cytological evaluation of all liquid-based FNACs and pathological diagnosis of thyroid specimens were conducted by an experienced cytopathologist (H.S.K.). Among 357 AUS/FLUS nodules, 210 nodules (59%) were re-evaluated before surgery; 122 CNB, 76 with FNAC and CNB, and 12 with FNAC. CNB and/or repeated FNAC showed 85 follicular neoplasm or suspicious for a follicular neoplasm (40%), 68 follicular lesion with nuclear/architectural atypia (33%), 46 malignant (22%), and 11 benign (5%). The CNB reporting system is based on the pathology reporting system proposed by the Korean Endocrine Pathology Thyroid Core Needle Biopsy Study Group (15). The majority of patients who had been referred to our hospital, a tertiary medical center, due to the result of AUS/FLUS on their initial FNAC outside the hospital underwent thyroid surgery because of inconclusive results of FNAC twice. Thyroid surgery was recommended for the indicated patients according to current guidelines (4, 16).

CLT was defined based on the pathologic report after thyroid surgery. Pathologic diagnosis of CLT is characterized by interstitial infiltration of hematopoietic mononuclear cells, mainly composed of lymphocytes with some plasma cells and macrophages. Lymphocytes penetrate the cytoplasm of thyrocytes and variable degrees of fibrosis are observed in the interstitium. Lesions of thyrocytes vary in intensity from atrophic thyrocytes to enlarged and bold thyrocytes (17). Tumor associated lymphoid infiltrates (TAICs) is histologically different from CLT in terms of the absence of Hurthle cell change of follicular cells. We separated CLT and TAICs in our surgical specimens by reading background thyroid remote from tumor and applying criteria that CLT is diffuse lymphocytic infiltration with Hurthle cell change; TAICs with absent Hurthle cell change. We excluded TAICs from this study.

### Laboratory Test for Thyroid Function

Serum thyrotropin (TSH, reference range, 0.55–4.78 mU/L), free thyroxine (FT4, reference range, 0.89–1.76 ng/dL), and triiodothyronine (T3, reference range, 60–181 ng/dL), were measured using a chemiluminescence immunoassay (Siemens Advia Centaur XP, Siemens Healthcare Diagnostics Inc., Tarrytown, NY, USA). The test sensitivities were 0.008 mU/L, 0.1 ng/dL, and 0.1 ng/dL. The interassay coefficients of variation (CV) were < 5%, < 5%, and < 2%, and intraassay CVs were < 5%, < 4%, and < 4%. Anti-thyroid peroxidase (anti-TPO) antibody and anti-thyroglobulin (anti-TG) antibody levels (reference range, 0–60 U/mL) were measured using a radioimmunoassay kit (B.R.A.H.M.S. GmbH, Hennigsdorf, Germany); the sensitivity was 5.5 U/mL, the interassay CVs were < 6% and < 10%, and intr-assay CVs were < 8% and < 5%. Titers of anti-TPO antibody and anti-TG antibody were measured in 138 (39%) and 210 (59%) patients, respectively.

### Statistical Analyses

Statistical analyses were performed using SPSS Statistics 18 (SPSS Inc., Chicago, IL, USA). Descriptive statistics (mean, standard deviation (SD), number, and percentage) were tabulated for baseline characteristics. The independent *t*-test was used for parametric measures, and a chi-square test was used to compare categorical variables. Cox regression analysis was used to identify whether CLT and other variables were independent risk factors for thyroid malignancies. A P value < 0.05 was considered statistically significant.

## Results

### Baseline Characteristics

Among 357 specimens, 130 tumors (36%) were confirmed to have coexisting CLT after thyroid surgery. Both AUS/FLUS groups with or without CLT showed comparable baseline parameters, including age, BMI, distribution of K-TIRADS, tumor multiplicity, rate of *BRAF* ^V600E^ mutation, and extent of thyroidectomy (**Table 1**). However, the AUS/FLUS with CLT group had a higher percentage of females (88% *vs.* 71%), smaller tumors (mean size, 1.9 cm *vs.* 2.4 cm), frequent diffuse or focal thyroiditis on US (39% *vs.* 14%), lymphocytic thyroiditis background on FNAC (18% *vs.* 4%), higher concentrations of serum TSH (mean, 2.87 mU/L *vs.* 1.65 mU/L), anti-TPO antibody (mean, 905 U/mL *vs.* 68 U/mL), and anti-TG antibody (mean, 180 U/mL *vs.* 19 U/mL), and a higher rate of thyroid medication at baseline (9% *vs.* 3%) than the AUS/FLUS without CLT group.

**Table 1:** Baseline characteristics of 357 Bethesda class III thyroid nodules.

Variables	With chronic lymphocytic thyroiditis (n = 130)	Without chronic lymphocytic thyroiditis (n = 227)	P-value
Age, years	51 ± 12	52 ± 13	0.58
Sex, female	114 (88%)	161 (71%)	**<0.001**
BMI, kg/m^2^	24.5 ± 3.9	24.7 ± 3.8	0.63
Thyroid nodule size, cm	1.9 ± 1.4	2.4 ± 1.7	**0.004**
Thyroid US finding, K-TIRADS			0.30
Benign	1 (1%)	2 (1%)	
Low suspicion	25 (19%)	62 (27%)	
Intermediate suspicion	73 (56%)	124 (55%)	
High suspicion	31 (24%)	39 (17%)	
Thyroiditis on US imaging	51 (39%)	31 (14%)	**<0.001**
Multiplicity	93 (72%)	169 (74%)	0.37
FNAC finding			
Hürthle cell change	17 (13%)	18 (8%)	0.12
Lymphocytic thyroiditis background	24 (18%)	10 (4%)	**<0.001**
*BRAF* ^V600E^ mutation	4/76 (5%)	11/130 (8%)	0.58
Preoperative thyroid function			
T3, ng/dL	101 ± 33	103 ± 22	0.49
FT4, ng/dL	1.17 ± 0.27	1.19 ± 0.22	0.50
TSH, mU/L	2.87 ± 5.07	1.65 ± 1.18	**0.008**
TSH above references	7 (5%)	8 (4%)	0.22
Anti-TPO antibody, U/mL	905 ± 1216	68 ± 345	**<0.001**
Anti-TPO above references	33/62 (53%)	2/76 (3%)	**<0.001**
Anti-TG antibody, U/mL	180 ± 431	19 ± 18	**0.001**
Anti-TG above references	31/79 (39%)	5/131 (4%)	**<0.001**
Both antibodies above references	12/62 (19%)	1/76 (1%)	**<0.001**
Thyroid medication	12 (9%)	6 (3%)	**0.001**
Levothyroxine	8 (6%)	0	
ATD	4 (3%)	6 (3%)	
Thyroidectomy			0.25
Lobectomy	61 (47%)	129 (57%)	
TT	68 (52%)	96 (42%)	
TT with MRND	1 (1%)	2 (1%)	
Pathology			0.98
Benign	68 (52%)	119 (52%)	
Malignancy	62 (48%)	108 (48%)	

US, ultrasonography; K-TIRADS, the Korean Thyroid Imaging Reporting and Data System; FNAC, fine needle aspiration cytology; T3, triiodothyronine; FT4, free thyroxine; TSH, thyrotropin; anti-TPO antibody, anti-thyroid peroxidase antibody; anti-TG antibody, anti-thyroglobulin antibody; ATD, anti-thyroid drug; TT, total thyroidectomy; MRND, modified radical neck dissection.

Variables are presented as the mean ± standard deviation (SD) or number (percentage).

Upper reference for TSH, anti-TPO antibody, and anti-TG antibody was 4.78 mU/L, 60 U/mL, and 60 U/mL, respectively.

Titers of anti-TPO antibody and anti-TG antibody were measured in 138 and 210 patients, respectively.

Significant results (P < 0.05) are indicated in bold.

### Pathologic Results Based on the Presence of CLT

Among a total of 357 tumors, 170 malignant (48%) and 187 benign (52%) tumors were diagnosed after thyroidectomy. Almost all malignancies (98%) were differentiated thyroid carcinomas except for 4 medullary thyroid carcinomas (MTCs) and 2 anaplastic thyroid carcinomas (ATCs; **Table 2**). Among malignancies, follicular variant of PTC (FVPTC, 114 cases, 67%) was the most frequently diagnosed, followed by classic PTC (35 cases, 21%) and follicular thyroid carcinoma (FTC, 15 cases, 9%). The distribution of malignant pathology was similar in both AUS/FLUS groups regardless of the presence CLT.

**Table 2:** Detailed pathology of 357 Bethesda class III thyroid nodules based on the presence of chronic lymphocytic thyroiditis.

Pathology	With chronic lymphocytic thyroiditis (n = 130)	Without chronic lymphocytic thyroiditis (n = 227)	P-value
Benign	68	119	**0.003**
Follicular adenoma	34 (50%)	71 (60%)	
Hyperplastic nodule	26 (38%)	47 (39%)	
Thyroiditis	8 (12%)	1 (1%)	
Malignancy	62	108	0.37
Classic PTC	10 (16%)	25 (23%)	
FVPTC	44 (71%)	70 (65%)	
FTC	4 (7%)	11 (10%)	
miFTC	4	8	
wiFTC	0	2	
oFTC	0	1	
MTC	3 (5%)	1 (1%)	
ATC	1 (1%)	1 (1%)	

PTC, papillary thyroid carcinoma; FVPTC, follicular variant of PTC; FTC, follicular thyroid carcinoma; miFTC, minimally invasive FTC; wiFTC, widely invasive FTC; oFTC, oncocytic variant of FTC; MTC, medullary thyroid carcinoma; ATC, anaplastic thyroid carcinoma.

Significant results (P < 0.05) are indicated in bold.

Among benign pathologies, follicular adenoma (105 cases, 56%) was the most prevalent diagnosis, followed by hyperplastic nodules (73 cases, 39%). Notably, thyroiditis was more frequently diagnosed as the final pathology in the AUS/FLUS with CLT group than in the AUS/FLUS without CLT group (12% *vs.* 1%, P = 0.003).

### Risk Factors for Differentiated Thyroid Malignancies

Cox regression analysis was performed to identify risk factors for differentiated thyroid malignancies except for 4 MTCs and 2 ATCs (**Table 3**). In multivariate analysis, the positive result of *BRAF* ^V600E^ mutation (hazard ratio (HR) 3.19, 95% CI 1.32–7.72, P < 0.001), highly suspicious features (K-TIRADS 5) on US (HR 2.07, 95% CI 1.09–3.96, P = 0.04), and smaller thyroid nodules (HR 0.81, 95% CI 0.69–0.96, P = 0.01) were significant risk factors for differentiated thyroid malignancies; however, CLT was not significant (p = 1.02).

**Table 3:** Cox regression model for the risk factors for differentiated thyroid malignancies.

Variables	Univariate		Multivariate	
	P-value	HR (95% CI)	P-value	HR (95% CI)
Age	0.38	0.99 (0.98–1.01)	0.64	0.99 (0.98–1.01)
Sex	0.29	1.32 (0.79–2.19)	0.22	1.14 (0.81–2.45)
BMI	0.43	1.02 (0.96–1.08)	0.56	1.02 (0.96–1.09)
Serum TSH	0.45	0.97 (0.91–1.05)	0.40	0.96 (0.88–1.05)
Thyroid nodule size	**<0.001**	**0.74 (0.63–0.86)**	**0.01**	**0.81 (0.69–0.96)**
Thyroid US finding, K-TIRADS 5	**<0.001**	**3.49 (1.97–6.18)**	**0.04**	**2.07 (1.09–3.96)**
Multiplicity	0.32	1.28 (0.79–2.07)	0.47	1.54 (0.88–2.69)
*BRAF* ^V600E^ mutation	**<0.001**	**3.32 (1.43–7.97)**	**<0.001**	**3.19 (1.32–7.72)**
Chronic lymphocytic thyroiditis	0.88	0.97 (0.62–1.50)	0.94	1.02 (0.61–1.69)
Thyroid medication	0.50	1.39 (0.53–3.68)	0.69	1.24 (0.43–3.60)

HR, hazard ratio; CI, confidence interval; TSH, thyrotropin; US, ultrasonography; K-TIRADS, the Korean Thyroid Imaging Reporting and Data System.

Cox regression analysis was performed except for 4 medullary and 2 anaplastic thyroid carcinomas.

Significant results (P < 0.05) are indicated in bold.

## Discussion

Preoperative diagnosis and management of Bethesda class III thyroid nodules are challenging for clinicians despite various efforts to assess the malignancy risk of thyroid nodules. Various pathologic changes of the thyroids caused by HT may lead to interference in the preoperative diagnosis of thyroid nodules with AUS/FLUS cytology. In addition, an association between chronic inflammation caused by HT and thyroid malignancy has been suggested. In the present study, surgically resected thyroid specimens were evaluated and the coexistence of CLT was determined to not affect the malignancy rate of thyroid nodules with AUS/FLUS cytology.

The Bethesda III is an inhomogenous category with various cytological features and CLT also show various pathologic changes in thyroids with the disease progression. AUS/FLUS includes mainly sparse and compromised samples with focally enlarged nuclei and atypical lymphoid infiltrate, features suggestive of PTC in a sample predominantly appearing benign (18) however this category is insufficient for diagnosis as follicular neoplasm or suspicious for malignancy (3). CLT caused by HT is characterized by epithelial changes and variable atypia (19). Microscopic findings of early CLT include enlarged nuclei, lymphocytic and plasma cell infiltration, atrophic follicles with abundant oncocytes, and atypical epithelium (10). The microscopic features of progressed CLT include fibrosis and nodularity or atrophic parenchyma, which are distinct from AUS/FLUS (10).

In addition, CLT may be associated with neoplastic changes in the thyroid glands. Two hypotheses have been proposed although an exact mechanism between CLT and malignancy is not fully understood. One explanation is that autoimmune infiltration of thyroid glands causes cellular damage and further changes the development of thyroid malignancy (20, 21). Another hypothesis is the association between elevated TSH and the increased risk of thyroid malignancy (22). In a meta-analysis by Lai et al. (11), the overall PTC risk in HT populations was higher than that in non-HT populations (OR 2.12), although the mean rate of PTC in HT populations ranged widely from 1% (selective FNA or thyroidectomy studies) to 40% (thyroidectomy studies) (11). In addition, the risk was higher in the Asian population (OR 2.79) than in American (OR 1.92) or European (OR 1.56) populations (11) and was probably associated with the higher prevalence of HT in iodine-sufficient areas (23, 24). Recently, HT-related atypia was named follicular epithelial dysplasia (FED) by Chui et al. (25) and was suggested to be a premalignant precursor of PTC (19).

HT influences the malignancy risk of thyroid nodules with AUS/FLUS cytology in two different ways. HT may complicate the accurate diagnosis of thyroid nodules. In another aspect, HT can be a premalignant condition and truly increase the risk of malignancy.

However, in clinical studies, whether coexisting CLT is associated with a higher risk of thyroid malignancy remains controversial. Mulder et al. (10) reported a lower rate of malignancy in AUS/FLUS cytology coexisting with CLT (44%, 32/73 nodules) than without CLT (60%, 131/220 nodules) in the US population (P = 0.02). The authors suggested that atypia due to CLT may falsely increase the diagnosis of AUS/FLUS; thus, AUS/FLUS cytology with a CLT background shows a lower prevalence of thyroid malignancy. However, in two previous studies conducted in US (26) and Korean (27) populations, CLT was not a predictor of thyroid malignancy. Wong et al. used 576 Hürthle cell-predominant FNAC samples and reported a comparable rate of CLT in benign (25%, 116/455 nodules) and malignant nodules (18%, 22/121 nodules; P = 0.12) (26). Suh et al. examined 446 nodules with AUS/FLUS cytology and reported that CLT was not a risk factor for thyroid malignancy. Similar to the present study results, Suh et al. concluded that US findings with highly suspicious features (K-TIRADS 5) (OR 11.02) and positive *BRAF* ^V600E^ mutation (OR 4.54) were significant factors for thyroid malignancies (27). In agreement with studies by Wong (26) and Suh et al. (27), CLT was not a risk factor for thyroid malignancies in AUS/FLUS cytology in the present study, unlike potent radiologic (highly suspicious features on US) and molecular (*BRAF* ^V600E^ mutation) predictors of thyroid malignancy.

In the present study, only surgically resected thyroid tumors were included because the pathologic definition is the preferred gold standard for CLT. Although concomitant malignant nodules were excluded, the malignancy rate of thyroid nodules (48%) in this study was relatively higher than the implied risk suggested by the Bethesda System for Reporting Thyroid Cytopathology (TBSRTC) (3) and comparable with the risk reported in studies using thyroidectomized specimens (5–7), as expected. In addition, FVPTC was the dominant pathology (61%) in malignancies, and the positive rate of *BRAF* ^V600E^ mutation was low (4% of all tumors) because thyroid nodules with AUS/FLUS cytology were investigated. In the literature, *RAS* is the most prevalent mutation and FVPTC, rather than classic PTC, is the most common malignancy in nodules with indeterminate cytology (28, 29).

The present study had several limitations. First, due to the retrospective design of the study, bias may have existed despite statistical correction. Second, selection bias may have occurred because only surgically resected specimens were included. Nevertheless, background HT is not a determinant factor for clinicians to make decisions regarding thyroid surgery, and all specimens are surgically resected; thus, biases from specimens, whether they were surgically resected or not, do not distort the results. Third, various molecular tests other than *BRAF* ^V600E^ were not conducted because only the *BRAF* ^V600E^ test was available for routine clinical practice in our institution for most of the study period. Molecular testing may aid in the prediction of thyroid malignancy; however, this was beyond the scope of this study.

In the present study, the malignancy rate of thyroid nodules with AUS/FLUS cytology was similar irrespective of the presence of background CLT.

## Data Availability Statement

The data supporting the conclusions of this article will be made available by the corresponding author, without undue reservation.

## Ethics Statement

The studies involving human participants were reviewed and approved by The Institutional Review Board of the Chung-Ang University Hospital. Written informed consent for participation was not required for this study in accordance with the national legislation and the institutional requirements.

## Author Contributions

YJC designed the research. YJC and HSK led sample collection. YYC analyzed the data and led the manuscript writing. YJC and YYC produced maps and final data tables. All authors contributed to the article and approved the submitted version.

## Funding

This work was partly supported by the Soonchunhyang University Research Fund.

## Conflict of Interest

The authors declare that the research was conducted in the absence of any commercial or financial relationships that could be construed as a potential conflict of interest.

## Publisher’s Note

All claims expressed in this article are solely those of the authors and do not necessarily represent those of their affiliated organizations, or those of the publisher, the editors and the reviewers. Any product that may be evaluated in this article, or claim that may be made by its manufacturer, is not guaranteed or endorsed by the publisher.

## References

[1] Guth S Theune U Aberle J Galach A Bamberger CM. Very High Prevalence of Thyroid Nodules Detected by High Frequency (13 MHz) Ultrasound Examination. Eur J Clin Invest (2009) 39:699–706. doi: 10.1111/j.1365-2362.2009.02162.x 19601965

[2] Tan GH Gharib H. Thyroid Incidentalomas: Management Approaches to Nonpalpable Nodules Discovered Incidentally on Thyroid Imaging. Ann Intern Med (1997) 126:226–31. doi: 10.7326/0003-4819-126-3-199702010-00009 9027275

[3] Cibas ES Ali SZ. The 2017 Bethesda System for Reporting Thyroid Cytopathology. Thyroid (2017) 27:1341–6. doi: 10.1089/thy.2017.0500 29091573

[4] Haugen BR Alexander EK Bible KC Doherty GM Mandel SJ Nikiforov YE. American Thyroid Association Management Guidelines for Adult Patients With Thyroid Nodules and Differentiated Thyroid Cancer: The American Thyroid Association Guidelines Task Force on Thyroid Nodules and Differentiated Thyroid Cancer. Thyroid (2016) 26:1–133. doi: 10.1089/thy.2015.0020 26462967 PMC4739132

[5] Lee YB Cho YY Jang JY Kim TH Jang HW Chung JH. Current Status and Diagnostic Values of the Bethesda System for Reporting Thyroid Cytopathology in a Papillary Thyroid Carcinoma-Prevalent Area. Head Neck (2017) 39:269–74. doi: 10.1002/hed.24578 27617626

[6] Turkyilmaz S Ulusahin M Celebi B Cekic AB Mungan S Kucuktulu U. Thyroid Nodules Classified as Atypia or Follicular Lesions of Undetermined Significance Deserve Further Research: Analysis of 305 Surgically Confirmed Nodules. Cytopathology (2017) 28:391–9. doi: 10.1111/cyt.12438 28714532

[7] Linhares SM Handelsman R Picado O Farrá JC Lew JI. Fine Needle Aspiration and the Bethesda System: Correlation With Histopathology in 1,228 Surgical Patients. Surgery (2021). doi: 10.1016/j.surg.2021.05.016 34134896

[8] Arena S Benvenga S. Gender-Specific Correlation of Intranodular Chronic Lymphocytic Thyroiditis With Thyroid Nodule Size, Echogenicity, and Histologically-Verified Cytological Class of Malignancy Risk. J Clin Transl Endocrinol (2018) 14:39–45. doi: 10.1016/j.jcte.2018.10.003 30416974 PMC6216079

[9] Bhatia A Rajwanshi A Dash RJ Mittal BR Saxena AK. Lymphocytic Thyroiditis–Is Cytological Grading Significant? A Correlation of Grades With Clinical, Biochemical, Ultrasonographic and Radionuclide Parameters. Cytojournal (2007) 4:10. doi: 10.1186/1742-6413-4-10 17470291 PMC1877811

[10] Mulder MB Khazeni KC Sussman MS Lew JI Farrá JC. Chronic Lymphocytic Thyroiditis May Lower Accuracy of AUS/FLUS Cytopathology in Surgical Patients. J Surg Res (2020) 245:244–8. doi: 10.1016/j.jss.2019.07.068 31421369

[11] Lai X Xia Y Zhang B Li J. Jiang Y. A Meta-Analysis of Hashimoto’s Thyroiditis and Papillary Thyroid Carcinoma Risk. Oncotarget (2017) 8:62414–24. doi: 10.18632/oncotarget.18620 PMC5617515 28977955

[12] Shin JH Baek JH Chung J Ha EJ Kim JH Lee YH. Ultrasonography Diagnosis and Imaging-Based Management of Thyroid Nodules: Revised Korean Society of Thyroid Radiology Consensus Statement and Recommendations. Korean J Radiol (2016) 17:370–95. doi: 10.3348/kjr.2016.17.3.370 PMC4842857 27134526

[13] Kim PH Suh CH Baek JH Chung SR Choi YJ Lee JH. Unnecessary Thyroid Nodule Biopsy Rates Under Four Ultrasound Risk Stratification Systems: A Systematic Review and Meta-Analysis. Eur Radiol (2021) 31:2877–85. doi: 10.1007/s00330-020-07384-6 33057762

[14] Hong HS Lee JY. Diagnostic Performance of Ultrasound Patterns by K-TIRADS and 2015 ATA Guidelines in Risk Stratification of Thyroid Nodules and Follicular Lesions of Undetermined Significance. AJR Am J Roentgenol (2019) 213:444–50. doi: 10.2214/ajr.18.20961 31039023

[15] Jung CK Min HS Park HJ Song DE Kim JH Park SY. Pathology Reporting of Thyroid Core Needle Biopsy: A Proposal of the Korean Endocrine Pathology Thyroid Core Needle Biopsy Study Group. J Pathol Transl Med (2015) 49:288–99. doi: 10.4132/jptm.2015.06.04 PMC4508566 26081825

[16] Leenhardt L Erdogan MF Hegedus L Mandel SJ Paschke R Rago T. European Thyroid Association Guidelines for Cervical Ultrasound Scan and Ultrasound-Guided Techniques in the Postoperative Management of Patients With Thyroid Cancer. Eur Thyroid J (2013) 2:147–59. doi: 10.1159/000354537 PMC4017749 24847448

[17] Caturegli P De Remigis A Rose NR. Hashimoto Thyroiditis: Clinical and Diagnostic Criteria. Autoimmun Rev (2014) 13:391–7. doi: 10.1016/j.autrev.2014.01.007 24434360

[18] Kholová I Ludvíková M. Thyroid Atypia of Undetermined Significance or Follicular Lesion of Undetermined Significance: An Indispensable Bethesda 2010 Diagnostic Category or Waste Garbage? Acta Cytol (2014) 58:319–29. doi: 10.1159/000366498 25195864

[19] Kholová I Kalfert D Lintusaari J Rajakorpi E Ludvíková M. Follicular Epithelial Dysplasia as Hashimoto Thyroiditis-Related Atypia: A Series of 91 Specimens. Endocr Pathol (2021) 32(3):368–74. doi: 10.1007/s12022-021-09679-w PMC8370905 33991306

[20] Tamimi DM. The Association Between Chronic Lymphocytic Thyroiditis and Thyroid Tumors. Int J Surg Pathol (2002) 10:141–6. doi: 10.1177/106689690201000207 12075407

[21] Okayasu I Fujiwara M Hara Y Tanaka Y Rose NR. Association of Chronic Lymphocytic Thyroiditis and Thyroid Papillary Carcinoma. A Study of Surgical Cases Among Japanese, and White and African Americans. Cancer (1995) 76:2312–8. doi: 10.1002/1097-0142(19951201)76:11<2312::aid-cncr2820761120>3.0.co;2-h 8635037

[22] Fiore E Rago T Latrofa F Provenzale MA Piaggi P Delitala A. Hashimoto’s Thyroiditis Is Associated With Papillary Thyroid Carcinoma: Role of TSH and of Treatment With L-Thyroxine. Endocr Relat Cancer (2011) 18:429–37. doi: 10.1530/erc-11-0028 21565972

[23] Kim S Kwon YS Kim JY Hong KH Park YK. Association Between Iodine Nutrition Status and Thyroid Disease-Related Hormone in Korean Adults: Korean National Health and Nutrition Examination Survey VI (2013–2015). Nutrients (2019) 11:2757. doi: 10.3390/nu11112757 PMC6893705 31766270

[24] Teng W Shan Z Teng X Guan H Li Y Teng D. Effect of Iodine Intake on Thyroid Diseases in China. N Engl J Med (2006) 354:2783–93. doi: 10.1056/NEJMoa054022 16807415

[25] Chui MH Cassol CA Asa SL Mete O. Follicular Epithelial Dysplasia of the Thyroid: Morphological and Immunohistochemical Characterization of a Putative Preneoplastic Lesion to Papillary Thyroid Carcinoma in Chronic Lymphocytic Thyroiditis. Virchows Arch (2013) 462:557–63. doi: 10.1007/s00428-013-1397-1 23532502

[26] Wong KS Jo VY Lowe AC Faquin WC Renshaw AA Shah AA. Malignancy Risk for Solitary and Multiple Nodules in Hürthle Cell-Predominant Thyroid Fine-Needle Aspirations: A Multi-Institutional Study. Cancer Cytopathol (2020) 128:68–75. doi: 10.1002/cncy.22213 31751003 PMC7421467

[27] Suh YJ Choi YJ. Strategy to Reduce Unnecessary Surgeries in Thyroid Nodules With Cytology of Bethesda Category III (AUS/FLUS): A Retrospective Analysis of 667 Patients Diagnosed by Surgery. Endocrine (2020) 69:578–86. doi: 10.1007/s12020-020-02300-w 32297204

[28] Cho YY Park SY Shin JH Oh YL Choe JH Kim JH. Highly Sensitive and Specific Molecular Test for Mutations in the Diagnosis of Thyroid Nodules: A Prospective Study of BRAF-Prevalent Population. Int J Mol Sci (2020) 21:5629. doi: 10.3390/ijms21165629 PMC7460614 32781560

[29] Nikiforov YE Ohori NP Hodak SP Carty SE LeBeau SO Ferris RL. Impact of Mutational Testing on the Diagnosis and Management of Patients With Cytologically Indeterminate Thyroid Nodules: A Prospective Analysis of 1056 FNA Samples. J Clin Endocrinol Metab (2011) 96:3390–7. doi: 10.1210/jc.2011-1469 PMC3205883 21880806

